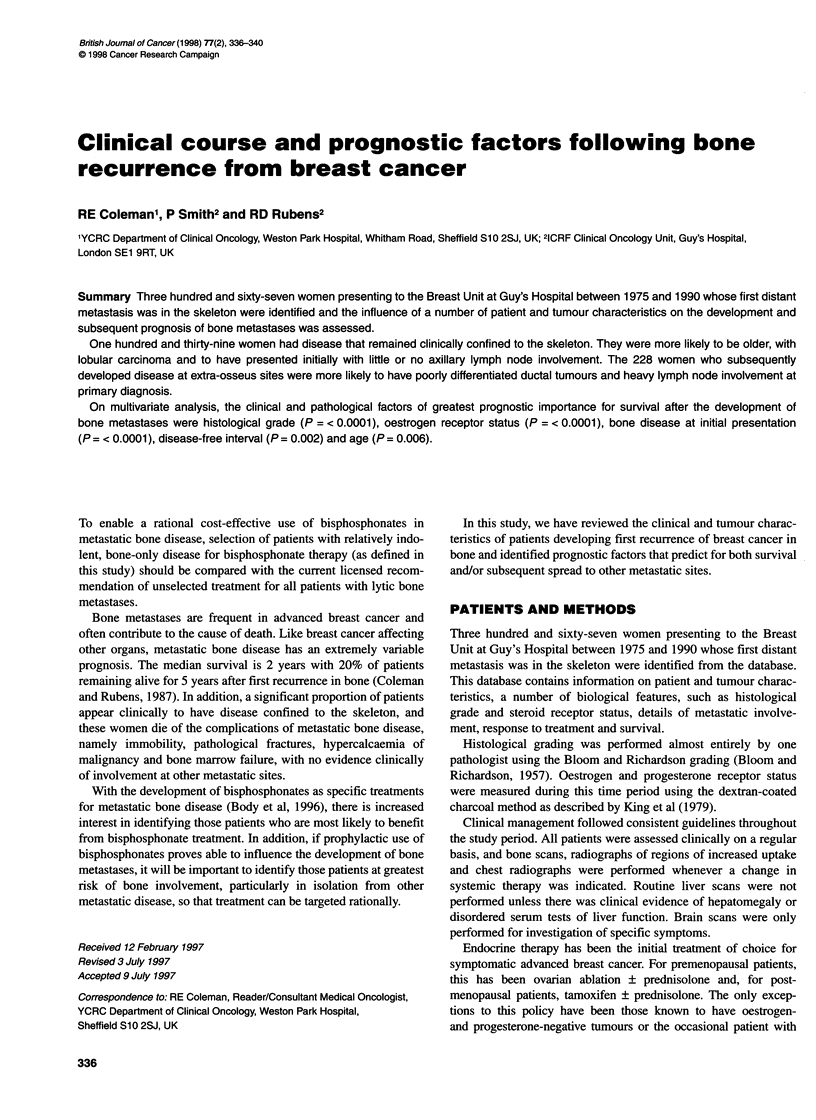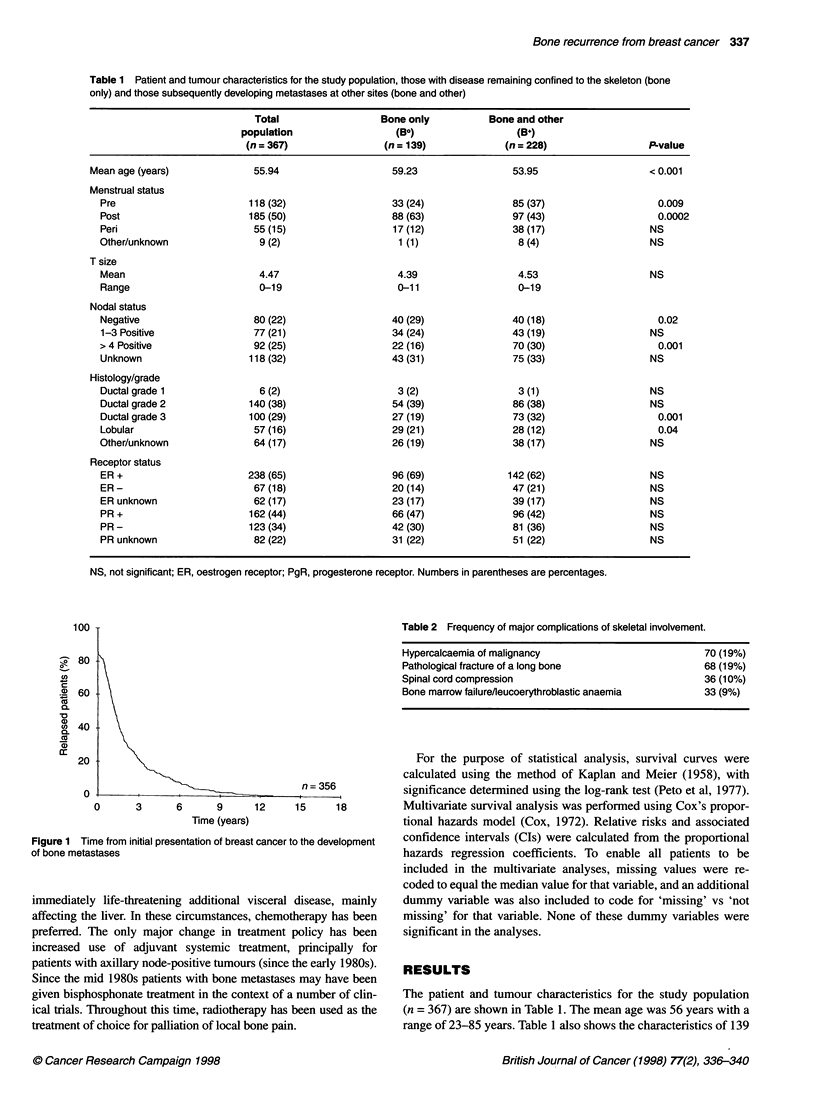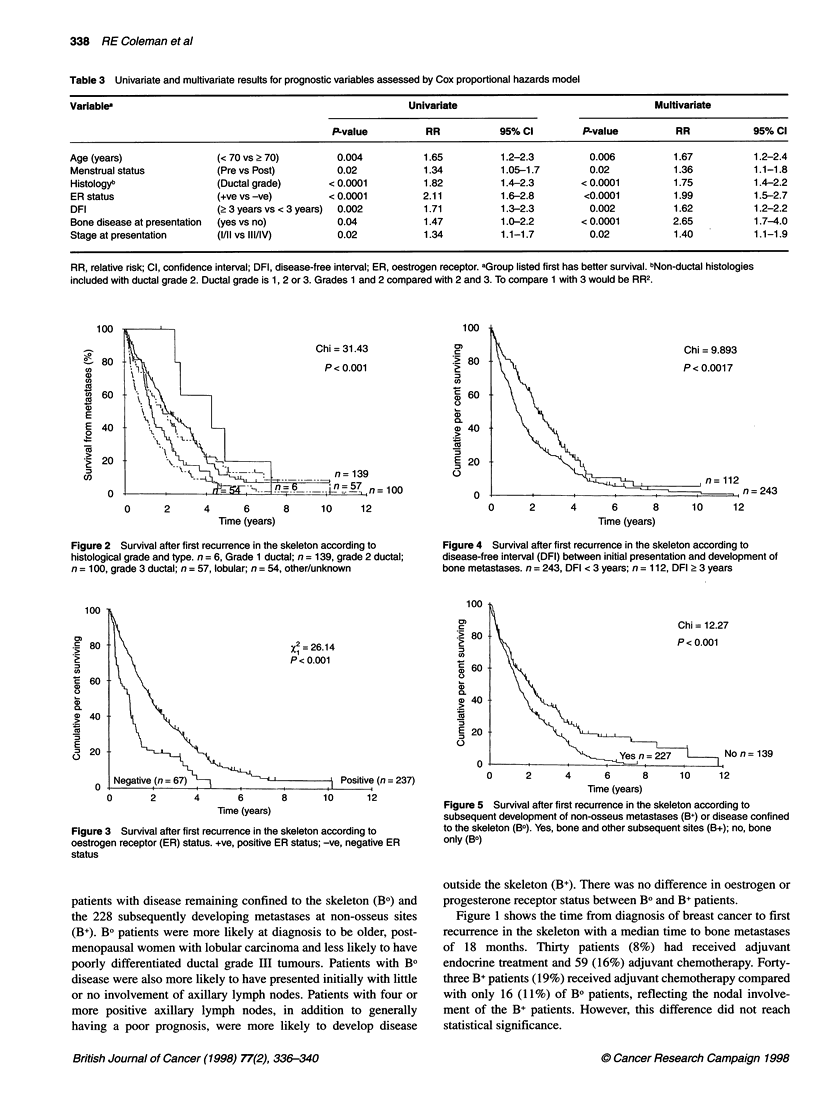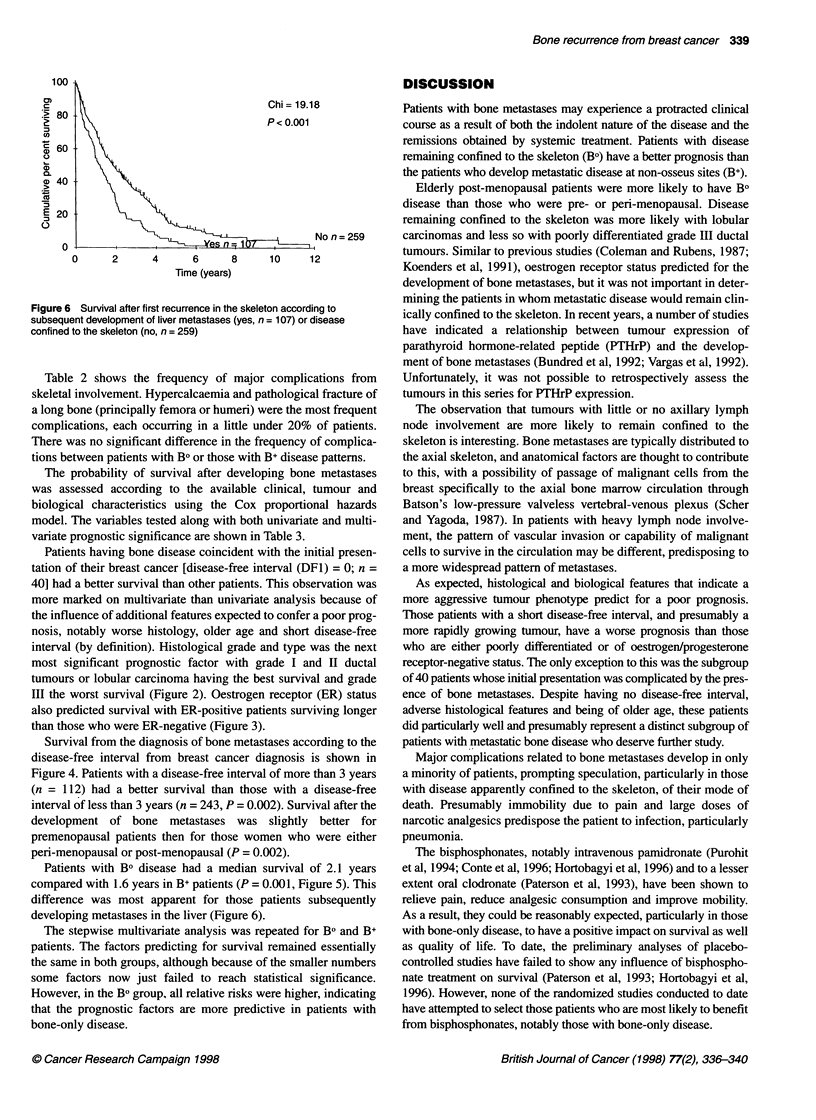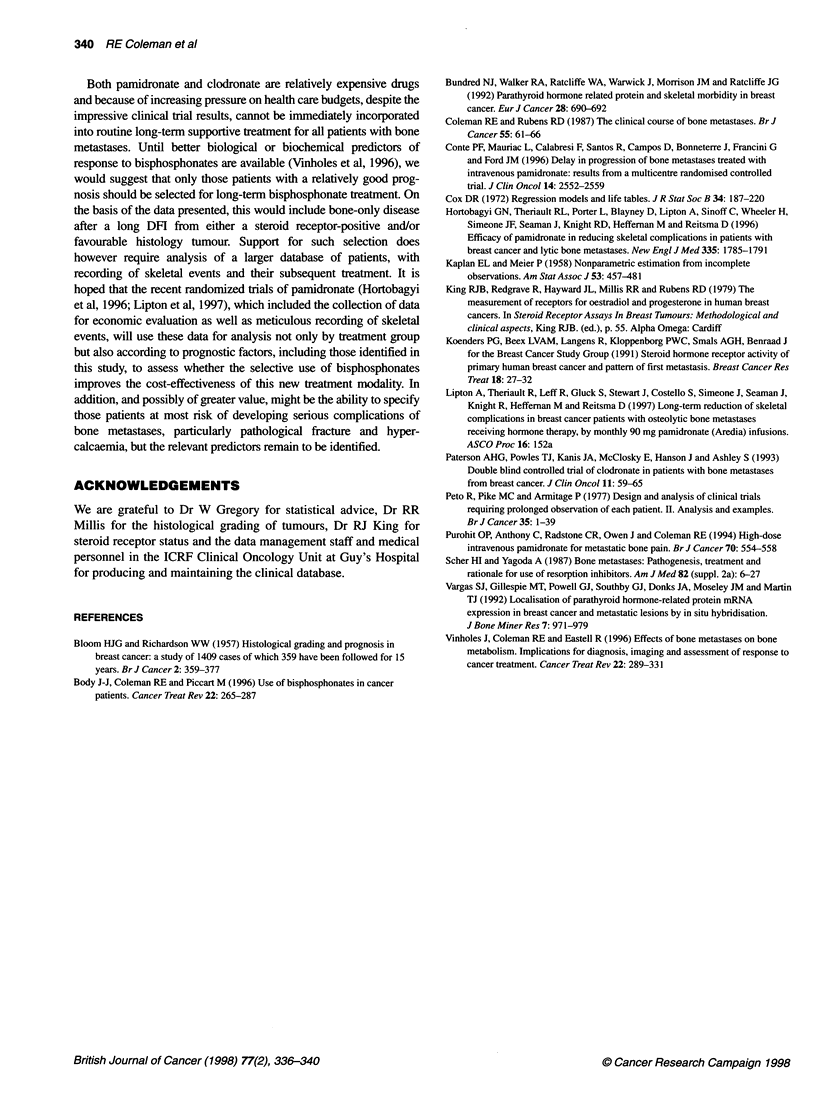# Clinical course and prognostic factors following bone recurrence from breast cancer.

**DOI:** 10.1038/bjc.1998.52

**Published:** 1998

**Authors:** R. E. Coleman, P. Smith, R. D. Rubens

**Affiliations:** YCRC Department of Clinical Oncology, Weston Park Hospital, Sheffield, UK.

## Abstract

Three hundred and sixty-seven women presenting to the Breast Unit at Guy's Hospital between 1975 and 1990 whose first distant metastasis was in the skeleton were identified and the influence of a number of patient and tumour characteristics on the development and subsequent prognosis of bone metastases was assessed. One hundred and thirty-nine women had disease that remained clinically confined to the skeleton. They were more likely to be older, with lobular carcinoma and to have presented initially with little or no axillary lymph node involvement. The 228 women who subsequently developed disease at extra-osseus sites were more likely to have poorly differentiated ductal tumours and heavy lymph node involvement at primary diagnosis. On multivariate analysis, the clinical and pathological factors of greatest prognostic importance for survival after the development of bone metastases were histological grade (P = < 0.0001), oestrogen receptor status (P = < 0.0001), bone disease at initial presentation (P = < 0.0001), disease-free interval (P = 0.002) and age (P = 0.006).


British Journal of Cancer (1998) 77(2), 336–340
© 1998 Cancer Research Campaign

### Clinical course and prognostic factors following bone recurrence from breast cancer

**RE Coleman**[1], **P Smith**[2] **and RD Rubens**[2]

[1]YCRC Department of Clinical Oncology, Weston Park Hospital, Whitham Road, Sheffield S10 2SJ, UK; [2]ICRF Clinical Oncology Unit, Guy's Hospital, London SE1 9RT, UK

**Summary** Three hundred and sixty-seven women presenting to the Breast Unit at Guy's Hospital between 1975 and 1990 whose first distant metastasis was in the skeleton were identified and the influence of a number of patient and tumour characteristics on the development and subsequent prognosis of bone metastases was assessed.

One hundred and thirty-nine women had disease that remained clinically confined to the skeleton. They were more likely to be older, with lobular carcinoma and to have presented initially with little or no axillary lymph node involvement. The 228 women who subsequently developed disease at extra-osseus sites were more likely to have poorly differentiated ductal tumours and heavy lymph node involvement at primary diagnosis.

On multivariate analysis, the clinical and pathological factors of greatest prognostic importance for survival after the development of bone metastases were histological grade ($P = < 0.0001$), oestrogen receptor status ($P = < 0.0001$), bone disease at initial presentation ($P = < 0.0001$), disease-free interval ($P = 0.002$) and age ($P = 0.006$).

To enable a rational cost-effective use of bisphosphonates in metastatic bone disease, selection of patients with relatively indolent, bone-only disease for bisphosphonate therapy (as defined in this study) should be compared with the current licensed recommendation of unselected treatment for all patients with lytic bone metastases.

Bone metastases are frequent in advanced breast cancer and often contribute to the cause of death. Like breast cancer affecting other organs, metastatic bone disease has an extremely variable prognosis. The median survival is 2 years with 20% of patients remaining alive for 5 years after first recurrence in bone (Coleman and Rubens, 1987). In addition, a significant proportion of patients appear clinically to have disease confined to the skeleton, and these women die of the complications of metastatic bone disease, namely immobility, pathological fractures, hypercalcaemia of malignancy and bone marrow failure, with no evidence clinically of involvement at other metastatic sites.

With the development of bisphosphonates as specific treatments for metastatic bone disease (Body et al, 1996), there is increased interest in identifying those patients who are most likely to benefit from bisphosphonate treatment. In addition, if prophylactic use of bisphosphonates proves able to influence the development of bone metastases, it will be important to identify those patients at greatest risk of bone involvement, particularly in isolation from other metastatic disease, so that treatment can be targeted rationally.

*Received 12 February 1997*
*Revised 3 July 1997*
*Accepted 9 July 1997*

*Correspondence to:* RE Coleman, Reader/Consultant Medical Oncologist, YCRC Department of Clinical Oncology, Weston Park Hospital, Sheffield S10 2SJ, UK

In this study, we have reviewed the clinical and tumour characteristics of patients developing first recurrence of breast cancer in bone and identified prognostic factors that predict for both survival and/or subsequent spread to other metastatic sites.

### PATIENTS AND METHODS

Three hundred and sixty-seven women presenting to the Breast Unit at Guy's Hospital between 1975 and 1990 whose first distant metastasis was in the skeleton were identified from the database. This database contains information on patient and tumour characteristics, a number of biological features, such as histological grade and steroid receptor status, details of metastatic involvement, response to treatment and survival.

Histological grading was performed almost entirely by one pathologist using the Bloom and Richardson grading (Bloom and Richardson, 1957). Oestrogen and progesterone receptor status were measured during this time period using the dextran-coated charcoal method as described by King et al (1979).

Clinical management followed consistent guidelines throughout the study period. All patients were assessed clinically on a regular basis, and bone scans, radiographs of regions of increased uptake and chest radiographs were performed whenever a change in systemic therapy was indicated. Routine liver scans were not performed unless there was clinical evidence of hepatomegaly or disordered serum tests of liver function. Brain scans were only performed for investigation of specific symptoms.

Endocrine therapy has been the initial treatment of choice for symptomatic advanced breast cancer. For premenopausal patients, this has been ovarian ablation ± prednisolone and, for postmenopausal patients, tamoxifen ± prednisolone. The only exceptions to this policy have been those known to have oestrogen- and progesterone-negative tumours or the occasional patient with

**Table 1** Patient and tumour characteristics for the study population, those with disease remaining confined to the skeleton (bone only) and those subsequently developing metastases at other sites (bone and other)

| | Total population (*n* = 367) | Bone only (B°) (*n* = 139) | Bone and other (B⁺) (*n* = 228) | *P*-value |
|---|---|---|---|---|
| Mean age (years) | 55.94 | 59.23 | 53.95 | < 0.001 |
| Menstrual status | | | | |
| Pre | 118 (32) | 33 (24) | 85 (37) | 0.009 |
| Post | 185 (50) | 88 (63) | 97 (43) | 0.0002 |
| Peri | 55 (15) | 17 (12) | 38 (17) | NS |
| Other/unknown | 9 (2) | 1 (1) | 8 (4) | NS |
| T size | | | | |
| Mean | 4.47 | 4.39 | 4.53 | NS |
| Range | 0–19 | 0–11 | 0–19 | |
| Nodal status | | | | |
| Negative | 80 (22) | 40 (29) | 40 (18) | 0.02 |
| 1–3 Positive | 77 (21) | 34 (24) | 43 (19) | NS |
| > 4 Positive | 92 (25) | 22 (16) | 70 (30) | 0.001 |
| Unknown | 118 (32) | 43 (31) | 75 (33) | NS |
| Histology/grade | | | | |
| Ductal grade 1 | 6 (2) | 3 (2) | 3 (1) | NS |
| Ductal grade 2 | 140 (38) | 54 (39) | 86 (38) | NS |
| Ductal grade 3 | 100 (29) | 27 (19) | 73 (32) | 0.001 |
| Lobular | 57 (16) | 29 (21) | 28 (12) | 0.04 |
| Other/unknown | 64 (17) | 26 (19) | 38 (17) | NS |
| Receptor status | | | | |
| ER + | 238 (65) | 96 (69) | 142 (62) | NS |
| ER – | 67 (18) | 20 (14) | 47 (21) | NS |
| ER unknown | 62 (17) | 23 (17) | 39 (17) | NS |
| PR + | 162 (44) | 66 (47) | 96 (42) | NS |
| PR – | 123 (34) | 42 (30) | 81 (36) | NS |
| PR unknown | 82 (22) | 31 (22) | 51 (22) | NS |

NS, not significant; ER, oestrogen receptor; PgR, progesterone receptor. Numbers in parentheses are percentages.

**Figure 1** Time from initial presentation of breast cancer to the development of bone metastases immediately life-threatening additional visceral disease, mainly affecting the liver. In these circumstances, chemotherapy has been preferred. The only major change in treatment policy has been increased use of adjuvant systemic treatment, principally for patients with axillary node-positive tumours (since the early 1980s). Since the mid 1980s patients with bone metastases may have been given bisphosphonate treatment in the context of a number of clinical trials. Throughout this time, radiotherapy has been used as the treatment of choice for palliation of local bone pain.

**Table 2** Frequency of major complications of skeletal involvement.

| | |
|---|---|
| Hypercalcaemia of malignancy | 70 (19%) |
| Pathological fracture of a long bone | 68 (19%) |
| Spinal cord compression | 36 (10%) |
| Bone marrow failure/leucoerythroblastic anaemia | 33 (9%) |

For the purpose of statistical analysis, survival curves were calculated using the method of Kaplan and Meier (1958), with significance determined using the log-rank test (Peto et al, 1977). Multivariate survival analysis was performed using Cox's proportional hazards model (Cox, 1972). Relative risks and associated confidence intervals (CIs) were calculated from the proportional hazards regression coefficients. To enable all patients to be included in the multivariate analyses, missing values were recoded to equal the median value for that variable, and an additional dummy variable was also included to code for 'missing' vs 'not missing' for that variable. None of these dummy variables were significant in the analyses.

### RESULTS

The patient and tumour characteristics for the study population ($n$ = 367) are shown in Table 1. The mean age was 56 years with a range of 23–85 years. Table 1 also shows the characteristics of 139

**Table 3** Univariate and multivariate results for prognostic variables assessed by Cox proportional hazards model

| Variable[a] | | Univariate | | | Multivariate | | |
|---|---|---|---|---|---|---|---|
| | | *P*-value | RR | 95% CI | *P*-value | RR | 95% CI |
| Age (years) | (< 70 vs ≥ 70) | 0.004 | 1.65 | 1.2–2.3 | 0.006 | 1.67 | 1.2–2.4 |
| Menstrual status | (Pre vs Post) | 0.02 | 1.34 | 1.05–1.7 | 0.02 | 1.36 | 1.1–1.8 |
| Histology[b] | (Ductal grade) | < 0.0001 | 1.82 | 1.4–2.3 | < 0.0001 | 1.75 | 1.4–2.2 |
| ER status | (+ve vs –ve) | < 0.0001 | 2.11 | 1.6–2.8 | <0.0001 | 1.99 | 1.5–2.7 |
| DFI | (≥ 3 years vs < 3 years) | 0.002 | 1.71 | 1.3–2.3 | 0.002 | 1.62 | 1.2–2.2 |
| Bone disease at presentation | (yes vs no) | 0.04 | 1.47 | 1.0–2.2 | < 0.0001 | 2.65 | 1.7–4.0 |
| Stage at presentation | (I/II vs III/IV) | 0.02 | 1.34 | 1.1–1.7 | 0.02 | 1.40 | 1.1–1.9 |

RR, relative risk; CI, confidence interval; DFI, disease-free interval; ER, oestrogen receptor. [a]Group listed first has better survival. [b]Non-ductal histologies included with ductal grade 2. Ductal grade is 1, 2 or 3. Grades 1 and 2 compared with 2 and 3. To compare 1 with 3 would be RR$^2$.

**Figure 2** Survival after first recurrence in the skeleton according to histological grade and type. *n* = 6, Grade 1 ductal; *n* = 139, grade 2 ductal; *n* = 100, grade 3 ductal; *n* = 57, lobular; *n* = 54, other/unknown

**Figure 4** Survival after first recurrence in the skeleton according to disease-free interval (DFI) between initial presentation and development of bone metastases. *n* = 243, DFI < 3 years; *n* = 112, DFI ≥ 3 years

**Figure 3** Survival after first recurrence in the skeleton according to oestrogen receptor (ER) status. +ve, positive ER status; –ve, negative ER status

**Figure 5** Survival after first recurrence in the skeleton according to subsequent development of non-osseus metastases (B$^+$) or disease confined to the skeleton (B$^o$). Yes, bone and other subsequent sites (B+); no, bone only (B$^o$)

patients with disease remaining confined to the skeleton (B$^o$) and the 228 subsequently developing metastases at non-osseus sites (B$^+$). B$^o$ patients were more likely at diagnosis to be older, post-menopausal women with lobular carcinoma and less likely to have poorly differentiated ductal grade III tumours. Patients with B$^o$ disease were also more likely to have presented initially with little or no involvement of axillary lymph nodes. Patients with four or more positive axillary lymph nodes, in addition to generally having a poor prognosis, were more likely to develop disease outside the skeleton (B$^+$). There was no difference in oestrogen or progesterone receptor status between B$^o$ and B$^+$ patients.

Figure 1 shows the time from diagnosis of breast cancer to first recurrence in the skeleton with a median time to bone metastases of 18 months. Thirty patients (8%) had received adjuvant endocrine treatment and 59 (16%) adjuvant chemotherapy. Forty-three B$^+$ patients (19%) received adjuvant chemotherapy compared with only 16 (11%) of B$^o$ patients, reflecting the nodal involvement of the B$^+$ patients. However, this difference did not reach statistical significance.

© Cancer Research Campaign 1998

**Figure 6** Survival after first recurrence in the skeleton according to subsequent development of liver metastases (yes, $n$ = 107) or disease confined to the skeleton (no, $n$ = 259)

Table 2 shows the frequency of major complications from skeletal involvement. Hypercalcaemia and pathological fracture of a long bone (principally femora or humeri) were the most frequent complications, each occurring in a little under 20% of patients. There was no significant difference in the frequency of complications between patients with B° or those with B+ disease patterns.

The probability of survival after developing bone metastases was assessed according to the available clinical, tumour and biological characteristics using the Cox proportional hazards model. The variables tested along with both univariate and multivariate prognostic significance are shown in Table 3.

Patients having bone disease coincident with the initial presentation of their breast cancer [disease-free interval (DF1) = 0; $n$ = 40] had a better survival than other patients. This observation was more marked on multivariate than univariate analysis because of the influence of additional features expected to confer a poor prognosis, notably worse histology, older age and short disease-free interval (by definition). Histological grade and type was the next most significant prognostic factor with grade I and II ductal tumours or lobular carcinoma having the best survival and grade III the worst survival (Figure 2). Oestrogen receptor (ER) status also predicted survival with ER-positive patients surviving longer than those who were ER-negative (Figure 3).

Survival from the diagnosis of bone metastases according to the disease-free interval from breast cancer diagnosis is shown in Figure 4. Patients with a disease-free interval of more than 3 years ($n$ = 112) had a better survival than those with a disease-free interval of less than 3 years ($n$ = 243, $P$ = 0.002). Survival after the development of bone metastases was slightly better for premenopausal patients then for those women who were either peri-menopausal or post-menopausal ($P$ = 0.002).

Patients with B° disease had a median survival of 2.1 years compared with 1.6 years in B+ patients ($P$ = 0.001, Figure 5). This difference was most apparent for those patients subsequently developing metastases in the liver (Figure 6).

The stepwise multivariate analysis was repeated for B° and B+ patients. The factors predicting for survival remained essentially the same in both groups, although because of the smaller numbers some factors now just failed to reach statistical significance. However, in the B° group, all relative risks were higher, indicating that the prognostic factors are more predictive in patients with bone-only disease.

### DISCUSSION

Patients with bone metastases may experience a protracted clinical course as a result of both the indolent nature of the disease and the remissions obtained by systemic treatment. Patients with disease remaining confined to the skeleton (B°) have a better prognosis than the patients who develop metastatic disease at non-osseus sites (B+).

Elderly post-menopausal patients were more likely to have B° disease than those who were pre- or peri-menopausal. Disease remaining confined to the skeleton was more likely with lobular carcinomas and less so with poorly differentiated grade III ductal tumours. Similar to previous studies (Coleman and Rubens, 1987; Koenders et al, 1991), oestrogen receptor status predicted for the development of bone metastases, but it was not important in determining the patients in whom metastatic disease would remain clinically confined to the skeleton. In recent years, a number of studies have indicated a relationship between tumour expression of parathyroid hormone-related peptide (PTHrP) and the development of bone metastases (Bundred et al, 1992; Vargas et al, 1992). Unfortunately, it was not possible to retrospectively assess the tumours in this series for PTHrP expression.

The observation that tumours with little or no axillary lymph node involvement are more likely to remain confined to the skeleton is interesting. Bone metastases are typically distributed to the axial skeleton, and anatomical factors are thought to contribute to this, with a possibility of passage of malignant cells from the breast specifically to the axial bone marrow circulation through Batson's low-pressure valveless vertebral-venous plexus (Scher and Yagoda, 1987). In patients with heavy lymph node involvement, the pattern of vascular invasion or capability of malignant cells to survive in the circulation may be different, predisposing to a more widespread pattern of metastases.

As expected, histological and biological features that indicate a more aggressive tumour phenotype predict for a poor prognosis. Those patients with a short disease-free interval, and presumably a more rapidly growing tumour, have a worse prognosis than those who are either poorly differentiated or of oestrogen/progesterone receptor-negative status. The only exception to this was the subgroup of 40 patients whose initial presentation was complicated by the presence of bone metastases. Despite having no disease-free interval, adverse histological features and being of older age, these patients did particularly well and presumably represent a distinct subgroup of patients with metastatic bone disease who deserve further study.

Major complications related to bone metastases develop in only a minority of patients, prompting speculation, particularly in those with disease apparently confined to the skeleton, of their mode of death. Presumably immobility due to pain and large doses of narcotic analgesics predispose the patient to infection, particularly pneumonia.

The bisphosphonates, notably intravenous pamidronate (Purohit et al, 1994; Conte et al, 1996; Hortobagyi et al, 1996) and to a lesser extent oral clodronate (Paterson et al, 1993), have been shown to relieve pain, reduce analgesic consumption and improve mobility. As a result, they could be reasonably expected, particularly in those with bone-only disease, to have a positive impact on survival as well as quality of life. To date, the preliminary analyses of placebo-controlled studies have failed to show any influence of bisphosphonate treatment on survival (Paterson et al, 1993; Hortobagyi et al, 1996). However, none of the randomized studies conducted to date have attempted to select those patients who are most likely to benefit from bisphosphonates, notably those with bone-only disease.

© Cancer Research Campaign 1998 

Both pamidronate and clodronate are relatively expensive drugs and because of increasing pressure on health care budgets, despite the impressive clinical trial results, cannot be immediately incorporated into routine long-term supportive treatment for all patients with bone metastases. Until better biological or biochemical predictors of response to bisphosphonates are available (Vinholes et al, 1996), we would suggest that only those patients with a relatively good prognosis should be selected for long-term bisphosphonate treatment. On the basis of the data presented, this would include bone-only disease after a long DFI from either a steroid receptor-positive and/or favourable histology tumour. Support for such selection does however require analysis of a larger database of patients, with recording of skeletal events and their subsequent treatment. It is hoped that the recent randomized trials of pamidronate (Hortobagyi et al, 1996; Lipton et al, 1997), which included the collection of data for economic evaluation as well as meticulous recording of skeletal events, will use these data for analysis not only by treatment group but also according to prognostic factors, including those identified in this study, to assess whether the selective use of bisphosphonates improves the cost-effectiveness of this new treatment modality. In addition, and possibly of greater value, might be the ability to specify those patients at most risk of developing serious complications of bone metastases, particularly pathological fracture and hypercalcaemia, but the relevant predictors remain to be identified.

### ACKNOWLEDGEMENTS

We are grateful to Dr W Gregory for statistical advice, Dr RR Millis for the histological grading of tumours, Dr RJ King for steroid receptor status and the data management staff and medical personnel in the ICRF Clinical Oncology Unit at Guy's Hospital for producing and maintaining the clinical database.

### REFERENCES

Bloom HJG and Richardson WW (1957) Histological grading and prognosis in breast cancer: a study of 1409 cases of which 359 have been followed for 15 years. *Br J Cancer* **2**: 359–377

Body J-J, Coleman RE and Piccart M (1996) Use of bisphosphonates in cancer patients. *Cancer Treat Rev* **22**: 265–287

Bundred NJ, Walker RA, Ratcliffe WA, Warwick J, Morrison JM and Ratcliffe JG (1992) Parathyroid hormone related protein and skeletal morbidity in breast cancer. *Eur J Cancer* **28**: 690–692

Coleman RE and Rubens RD (1987) The clinical course of bone metastases. *Br J Cancer* **55**: 61–66

Conte PF, Mauriac L, Calabresi F, Santos R, Campos D, Bonneterre J, Francini G and Ford JM (1996) Delay in progression of bone metastases treated with intravenous pamidronate: results from a multicentre randomised controlled trial. *J Clin Oncol* **14**: 2552–2559

Cox DR (1972) Regression models and life tables. *J R Stat Soc B* **34**: 187–220

Hortobagyi GN, Theriault RL, Porter L, Blayney D, Lipton A, Sinoff C, Wheeler H, Simeone JF, Seaman J, Knight RD, Heffernan M and Reitsma D (1996) Efficacy of pamidronate in reducing skeletal complications in patients with breast cancer and lytic bone metastases. *New Engl J Med* **335**: 1785–1791

Kaplan EL and Meier P (1958) Nonparametric estimation from incomplete observations. *Am Stat Assoc J* **53**: 457–481

King RJB, Redgrave R, Hayward JL, Millis RR and Rubens RD (1979) The measurement of receptors for oestradiol and progesterone in human breast cancers. In *Steroid Receptor Assays In Breast Tumours: Methodological and clinical aspects*, King RJB. (ed.), p. 55. Alpha Omega: Cardiff

Koenders PG, Beex LVAM, Langens R, Kloppenborg PWC, Smals AGH, Benraad J for the Breast Cancer Study Group (1991) Steroid hormone receptor activity of primary human breast cancer and pattern of first metastasis. *Breast Cancer Res Treat* **18**: 27–32

Lipton A, Theriault R, Leff R, Gluck S, Stewart J, Costello S, Simeone J, Seaman J, Knight R, Heffernan M and Reitsma D (1997) Long-term reduction of skeletal complications in breast cancer patients with osteolytic bone metastases receiving hormone therapy, by monthly 90 mg pamidronate (Aredia) infusions. *ASCO Proc* **16**: 152a

Paterson AHG, Powles TJ, Kanis JA, McClosky E, Hanson J and Ashley S (1993) Double blind controlled trial of clodronate in patients with bone metastases from breast cancer. *J Clin Oncol* **11**: 59–65

Peto R, Pike MC and Armitage P (1977) Design and analysis of clinical trials requiring prolonged observation of each patient. II. Analysis and examples. *Br J Cancer* **35**: 1–39

Purohit OP, Anthony C, Radstone CR, Owen J and Coleman RE (1994) High-dose intravenous pamidronate for metastatic bone pain. *Br J Cancer* **70**: 554–558

Scher HI and Yagoda A (1987) Bone metastases: Pathogenesis, treatment and rationale for use of resorption inhibitors. *Am J Med* **82** (suppl. 2a): 6–27

Vargas SJ, Gillespie MT, Powell GJ, Southby GJ, Donks JA, Moseley JM and Martin TJ (1992) Localisation of parathyroid hormone-related protein mRNA expression in breast cancer and metastatic lesions by in situ hybridisation. *J Bone Miner Res* **7**: 971–979

Vinholes J, Coleman RE and Eastell R (1996) Effects of bone metastases on bone metabolism. Implications for diagnosis, imaging and assessment of response to cancer treatment. *Cancer Treat Rev* **22**: 289–331

© Cancer Research Campaign 1998